# An In-Person and Online Intervention for Parkinson Disease (UPGRADE-PD): Protocol for a Patient-Centered and Culturally Tailored 3-Arm Crossover Trial

**DOI:** 10.2196/65490

**Published:** 2025-05-02

**Authors:** Michail Elpidoforou, Irene Grimani, Marianna Papadopoulou, Nikolaos Papagiannakis, Anastasia Bougea, Athina-Maria Simitsi, Evangelos Sfikas, Ioanna Alexandratou, Ioanna Alefanti, Roubina Antonelou, Christos Koros, Ioanna Mavroyianni, Chrysa Chrysovitsanou, Leonidas Stefanis, Daphne Bakalidou

**Affiliations:** 1 Laboratory of Neuromuscular and Cardiovascular Study of Motion – LANECASM Department of Physiotherapy University of West Attica Athens Greece; 2 Department of Health Data Processing Digital Governance Service Greek Ministry of Health Athens Greece; 3 Department of Physiotherapy University of West Attica Athens Greece; 4 1st Department of Neurology, Eginition Hospital Medical School National and Kapodistrian University of Athens Athens Greece

**Keywords:** Parkinson disease, dance, cultural tailoring, patient-centeredness, quality of life, frailty, sarcopenia

## Abstract

**Background:**

Dance for Parkinson’s Disease (DfPD) is a dance program for individuals with Parkinson disease (PD). There is a lack of knowledge about the effect of this program on frailty and sarcopenia experienced by patients with PD. In addition, no randomized controlled trial to date has investigated either the possible differential effects of in-person versus online DfPD or the possible effects of DfPD on clinical parameters in Greek patients with PD.

**Objective:**

We aimed to assess the efficacy, safety, and feasibility of a culturally tailored and patient-centered DfPD program offered both in-person and online to Greek patients with early- to midstage PD.

**Methods:**

This is a 3-arm crossover randomized controlled trial (in-person DfPD vs online DfPD vs control) of UPGRADE-PD (Upbeating Greek Application of Dance in Parkinson’s Disease). The experimental period will be 10 months, including three 2-month interventional periods of two 60-minute dance classes per week for each group (in-person DfPD vs online DfPD) versus a control group (nonintervention group), and two 2-month washout periods between each group for 40 Greek patients with early- to midstage PD. Assessments will be performed face-to-face at baseline and at the end of each study period and will include quality of life, fatigue, depressive symptoms, stress, anxiety, sarcopenia, frailty, balance, cognitive functions, movement and nonmovement PD symptoms, and BMI. Safety, feasibility, and patient satisfaction for each dance intervention (in-person DfPD vs online DfPD) will be assessed as well.

**Results:**

The study protocol was approved by the Medical Ethics Committee of the Eginition University Hospital in September 2022 and the Research and Ethics Committee of the University of West Attica in October 2023 and funded in September 2023. The first participant was enrolled in April 2023, and the trial is currently ongoing and will conclude in September 2024.

**Conclusions:**

The results of this study are expected to show the possible differential effect of a patient-centered and culturally tailored in-person vs online DfPD intervention on several movement and nonmovement symptoms, as well as on quality of life, sarcopenia, and frailty in people living with PD in Greece.

**Trial Registration:**

ClinicalTrials.gov NCT06220084; https://clinicaltrials.gov/study/NCT06220084

**International Registered Report Identifier (IRRID):**

DERR1-10.2196/65490

## Introduction

### Background

Parkinson disease (PD), an idiopathic, neurodegenerative, and progressive movement disorder, is accompanied by movement and nonmovement symptoms, such as bradykinesia, postural instability, and cognitive and mood disorders, which all negatively affect the quality of life (QoL) [1]. Furthermore, all the aforementioned PD symptoms seem to relate to the reduction of physical activity, muscle mass, and strength levels, as well as movement performance of patients with PD, thus increasing sarcopenia and frailty [2-4]. It is well accepted that systematic physical exercise enhances neuroplasticity [5]; prevents or delays frailty [4,6]; and improves symptoms such as balance, gait, and QoL in people with PD [7].

In dance, the human body and its purposefully selected movements are the basic tools for expression in various rhythmical and cultural contexts, whether artistic, educational, training, recreational, therapeutic, or religious [8,9]. As an art form expressed through movement, dance is a physical activity accompanied by musical stimuli that seems to activate the reward system [1] and has the potential to improve QoL of participants across various age groups [10], while the targeted use of auditory rhythmical information itself seems to be therapeutic for movement and nonmovement symptoms, as well as for QoL, in people with PD [11].

Humans, as social animals, have a fundamental need to belong [12], and dance-based interventions seem to reduce social isolation and loneliness among older people [13], while also facilitating their social inclusion [14], even during the social isolation caused by the COVID-19 pandemic [15]. Positive memories directly connected with dance experiences from a young age, as well as the supportive nature of dance, which enhances social interaction, and the emotional experiences that accompany the dance process, may be some of the reasons older adults prefer dance to other physical activities [16-18].

While physical exercise seems to induce neuroplasticity in people with PD [5], dance has been associated with favorable changes in the corticospinal tract, the corpus callosum, the superior longitudinal fasciculus, the dorsolateral prefrontal cortex, the bilateral putamen, the mirror neuron system, and sensorimotor pathways of healthy dancers [9,19]. Following the aforementioned effects of dance in neuroplasticity, dance in PD seems to enhance psychological flexibility (thus the person tends to adapt to environmental changes more efficiently) and creative self-efficacy (thus the person tends to think creatively and execute these thoughts) [20]. Dance, compared to other types of exercise, improves PD symptoms, such as balance, functional mobility, and cognition [21-24]. Moreover, dance is a substantial motivational means of exercise for patients with PD, enhancing their adherence rates to exercise programs [25,26], as well as a safe, feasible, and enjoyable intervention for patients with early- to midstage PD [27], even when the classes are remotely conducted [28].

Dance for Parkinson’s Disease (DfPD) is a structured dance program under appropriate care and supervision especially designed for people with PD, which integrates different dance genres [29]. Various studies have already described the content of the DfPD class that was followed [30-32]. To our knowledge, there is no published study investigating the effect of DfPD on the frailty and sarcopenia experienced by patients with PD. In addition, no randomized controlled trial to date has been conducted to investigate either the effect of this program on any clinical parameter in Greek patients with PD or the possible differential effect of in-person versus online DfPD. Furthermore, there is no published protocol of any patient-centered or culturally tailored dance intervention for the PD population showing the exact methodology that was followed and the content of the dance intervention. This study aims to fill the above gaps by publishing all the aforementioned data of our research protocol.

### Objective

The primary aim of this study is to investigate the possible effectiveness of a patient-centered and culturally tailored dance intervention delivered both in-person (in-person DfPD) and remotely (online DfPD) on QoL of Greek patients with early- to midstage PD. The key secondary aim is to investigate the safety and feasibility of the aforementioned interventions (ie, in-person DfPD and online DfPD) and its possible effectiveness on fatigue, depressive symptoms, stress, anxiety, sarcopenia, frailty, balance, cognitive functions, movement and nonmovement PD symptoms, and BMI of Greek patients with early- to midstage PD.

## Methods

### Study Design

**Figure 1:**
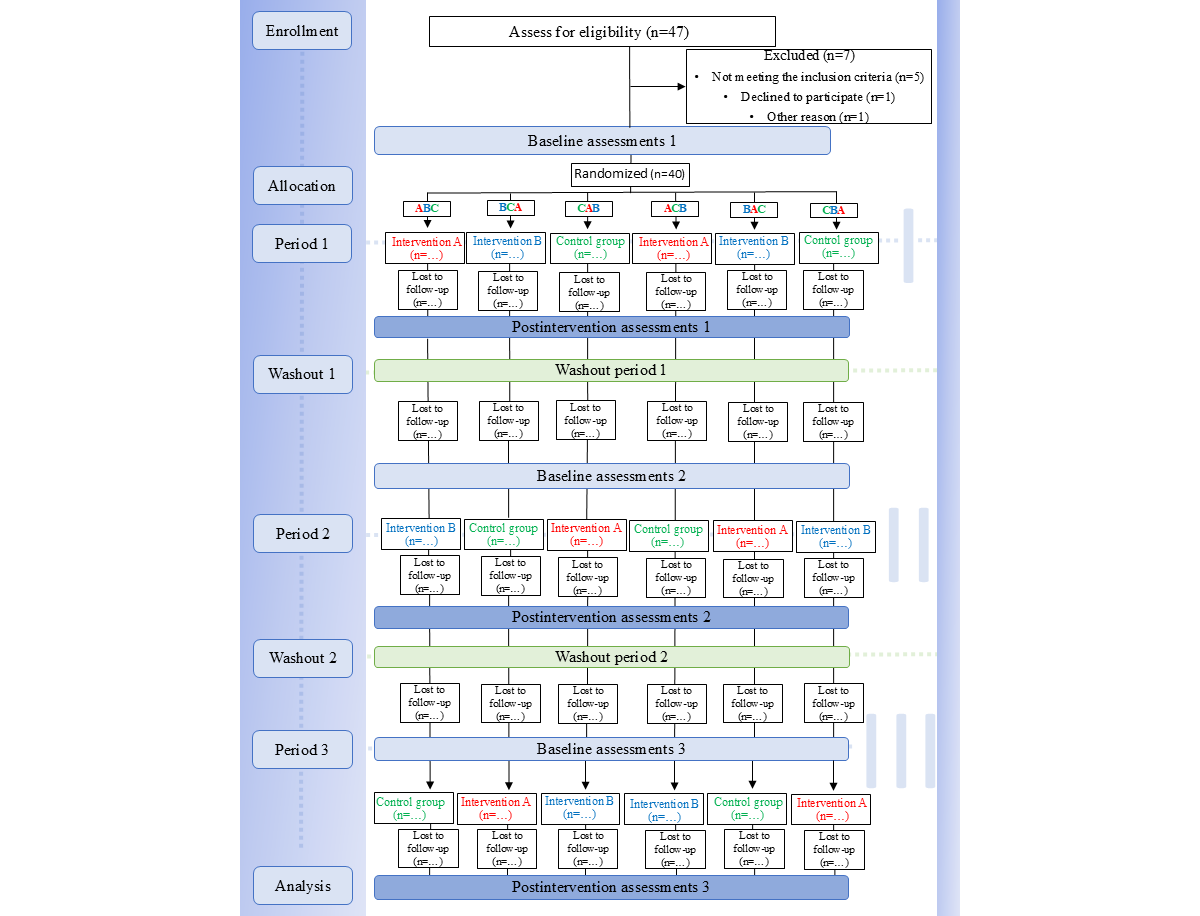
Flow diagram of the study.

This is a single-centered 3-arm crossover and open-label randomized controlled trial (in-person DfPD vs online DfPD vs control) of UPGRADE-PD (Upbeating Greek Application of Dance in Parkinson’s Disease). Blinding among participants and researchers is not possible due to the nature of the study. There are 6 possible research sequences, as shown in Table 1, and 3 possible carryover effects (1, 2, and 3) for each period from the prior intervention, as shown in Table 2. The study will last 10 months for each participant, including three 2-month research periods and two 2-month washout periods. Assessments will be conducted at enrollment (T_−0_=week −1), baseline periods (T_0_=week 0, T_2_=week 18, and T_4_=week 36), and postinterventional periods (T_1_=week 9, T_3_=week 27, and T_5_=week 45), as shown in Table 3. Allocation to each of the 6 possible sequences will occur after the completion of the first baseline assessment, as shown in the participant flow in Figure 1.

**Table 1:** The UPGRADE-PD (Upbeating Greek Application of Dance in Parkinson’s Disease) study design.

Sequence	Period		
	First	Second	Third
ABC	A^a^	B^b^	C^c^
BCA	B	C	A
CAB	C	A	B
ACB	A	C	B
BAC	B	A	C
CBA	C	B	A

^a^Intervention A (in-person Dance for Parkinson’s Disease combined with usual medical care).

^b^Intervention B (online Dance for Parkinson’s Disease combined with usual medical care).

^c^Control group (usual medical care without Dance for Parkinson’s Disease).

**Table 2:** The UPGRADE-PD (Upbeating Greek Application of Dance in Parkinson’s Disease) study’s code for the possible carryover effect.

Sequence	First period		Second period		Third period	
	Trial	Carryover effect	Trial	Carryover effect	Trial	Carryover effect
ABC	A^a^	0^b^	B^c^	1^d^	C^e^	2^f^
BCA	B	0	C	2	A	3^g^
CAB	C	0	A	3	B	1
ACB	A	0	C	1	B	3
BAC	B	0	A	2	C	1
CBA	C	0	B	3	A	2

^a^Intervention A (in-person Dance for Parkinson’s Disease combined with usual medical care).

^b^Noneffect.

^c^Intervention B (online Dance for Parkinson’s Disease combined with usual medical care).

^d^Effect from intervention A.

^e^Control group (usual medical care without Dance for Parkinson’s Disease).

^f^Effect from intervention B.

^g^Effect from the control group.

**Table 3:** The UPGRADE-PD (Upbeating Greek Application of Dance in Parkinson’s Disease) schedule of enrollment, interventions, and assessments for participants.

Time point (wk)		Study period									
		Enrollment (T_–0_=wk –1)	Baseline 1 (T_0_ =wk 0)	Allocation (T_0_ =wk 0)	Postintervention 1 (T_1_ =wk 9)	Wash-out 1 (T_1_ =wk 9)	Baseline 2 (T_2_ =wk 18)	Postintervention 2 (T_3_ =wk 27)	Wash-out 2 (T_3_ =wk 27)	Baseline 3 (T_4_ =wk 36)	Postintervention 3 (T_5_ =wk 45)
**Enrollment**											
	Eligibility screen	✓									
	Informed consent	✓									
	Allocation			✓							
**Interventions**											
	In-person DfPD^a^			A^b^	B^c^						
	Online DfPD						A	B			
	Control group									A	B
	Wash-out					A	B		A	B	
**Assessments**											
	SD^d^ and MH^e^		✓								
	AE^f^ and SAE^g^										✓
	Adherence rates										✓
	Attrition rates										✓
	Recruitment rates										✓
	Patient satisfaction										✓
	PDQ-8^h^		✓		✓		✓	✓		✓	✓
	PFS-16^i^		✓		✓		✓	✓		✓	✓
	MFIS^j^		✓		✓		✓	✓		✓	✓
	DASS-21^k^		✓		✓		✓	✓		✓	✓
	SARC-F^l^		✓		✓		✓	✓		✓	✓
	EWGSOP2^m^		✓		✓		✓	✓		✓	✓
	FP^n^		✓		✓		✓	✓		✓	✓
	BBS^o^		✓		✓		✓	✓		✓	✓
	MoCA^p^		✓		✓		✓	✓		✓	✓
	MDS-UPDRS^q^		✓		✓		✓	✓		✓	✓
	BMI		✓		✓		✓	✓		✓	✓
	Music preferences	✓									

^a^DfPD: Dance for Parkinson’s Disease.

^b^A: starting point of the period.

^c^B: ending point of the period.

^d^SD: sociodemographics.

^e^MH: medical history.

^f^AE: adverse event.

^g^SAE: serious adverse event.

^h^PDQ-8: Parkinson’s Disease Questionnaire-8.

^i^PFS-16: Parkinson Fatigue Scale-16.

^j^MFIS: Modified Fatigue Impact Scale.

^k^DASS-21: Depression, Anxiety, and Stress Scale-21.

^l^SARC-F: Strength, Assistance in walking, Rise from a chair, Climb stairs, and Falls Scale.

^m^EWGSOP2: European Working Group on Sarcopenia in Older People.

^n^FP: Frailty Phenotype.

^o^BBS: Berg Balance Scale.

^p^MoCA: Montreal Cognitive Assessment.

^q^MDS-UPDRS: Movement Disorder Society–sponsored revision of the Unified Parkinson’s Disease Rating Scale.

### Study Setting

We aim at examining whether following a twice per week DfPD class in-person or remotely for 8 weeks improves the study clinical parameters compared to usual care alone. A total of 40 patients with early- to midstage PD have been randomized to undergo a total of sixteen 60-minute DfPD classes twice weekly for more than 8 weeks, both in-person and online. The study will be conducted following the ethical principles of the Declaration of Helsinki and its later amendments [33], and the Pre-emptive Pharmacogenomic Testing for Preventing Adverse Drug Reactions (PREPARE) trial guide [34], and will conform with the CONSORT (Consolidated Standards of Reporting Trials) guidelines for randomized crossover trials [35], as shown in Figure 1, and the Standard Protocol Items Recommendations for Interventional Trials (SPIRIT) [36-38]. Eginition University Hospital Neurological Outpatient PD Clinic is the participating center, and the first participant was enrolled in April 2023.

### Ethical Considerations

The study protocol was approved by the Medical Ethics Committee of the Eginition University Hospital (approval 688/07.09.2022) and the Research and Ethics Committee of the University of West Attica (approval 92282/10/10/2023) and registered at ClinicalTrials.gov (NCT06220084). Written informed consent will be obtained from all participants. At the time of manuscript submission, recruitment has been concluded, but the trial is ongoing.

### Eligibility Criteria

The inclusion criteria for patients were set as follows: (1) age ≥18 years; (2) established diagnosis of idiopathic early to moderate PD, from 0 to 2.5 according to Hoehn and Yahr stages [39]; (3) being patient of the Eginition University Hospital Neurological Outpatient PD Clinic and under a stable medication for ≥4 weeks; (4) absence of other comorbidities or conditions that seriously affect or may affect mobility (eg, musculoskeletal or cardiovascular issues, pregnancy, terminal illnesses—such as cancer) according to attending physician’s discretion; (5) ability to understand, write, and speak in Greek; (6) written consent for participating in the study; (7) a consent of attending physician for participating in an exercise program, given they are totally aware of the patient’s clinical and physical status and medications; (8) smart device (either smartphone or tablet, laptop, or computer) access for the dance intervention that will be conducted remotely; (9) absence of serious cognitive impairment, according to attending physician’s discretion.

The exclusion criteria for patients were set as follows: (1) a non-PD tremor disorder diagnosis; (2) moderate to severe PD (≥3 Hoehn and Yahr stages) [39] because of a high fall risk [40]; (3) serious health or disability issues (either physical or mental, including pregnancy), because of which exercise is not permitted or exercise instructions cannot be followed; (4) hearing issues for which patient cannot follow music or verbal guidance; (5) non-PD related mental or mobility disorder; (6) any electronic internal medical device or implant, such as a pacemaker or a deep brain stimulation, due to contraindication in the use of bio-electrical impedance analysis [41]; (7) no access of the participant or patient’s carer to any smart device (either smart phone or tablet, laptop, or computer).

### Interventions

#### Explanation for the Choice of Comparators

The COVID-19 pandemic placed significant restrictions on the social circulation and exercise of patients with PD and highlighted the necessity of finding alternative forms of therapeutic exercise, such as telerehabilitation [42,43]. The use of telerehabilitation in neurological diseases is constantly expanding [44]. Online attendance in therapeutic dance classes for patients with PD is feasible and safe and is characterized by high compliance rates; it enables training of participants in new technologies and it is easy, fast, and inexpensive [27,28]. While we have already presented evidence that in-person DfPD is safe, feasible, and potentially effective in improving several movement and nonmovement symptoms of Greek patients with PD [45], there are no data regarding a related study conducted online.

#### Intervention Description

A total of sixteen 60-minute DfPD classes will be conducted twice weekly for 8 weeks, both in-person and online, in a physiotherapy facility in Athens, Greece (in-person DfPD), and via Zoom platform (online DfPD; Zoom Communications, Inc), which will be free for the participants; the facility is licensed for the establishment and operation of physiotherapy activities. All the dance classes will be instructed by the main investigator (ME), who holds the approval to use DfPD for research reasons and is a licensed neurological physiotherapist, adapted physical educator, and dance educator. The exact structure and content of the program are described in Table 4. Participants will be instructed individually on how to use the Zoom platform before their program entry. Frequency and duration of the program are in accordance with past exercise and dance programs in patients with PD [30,45-48].

**Table 4:** UPGRADE-PD (Upbeating Greek Application of Dance in Parkinson’s Disease) study’s DfPD structure and content.

Part (category) and track number		Type of activity		Type of exercise	Primal goals	Song title, album title (release date), composer
**1 (seated warm-up)**						
	1	Introduction: “The Sun Salutation”	Breathing exercises and gradual early mobilization		General warm-up, increase of concentration, and general mobilization	“Love in the Heart” (remastered 2004), *30 Nocturnes* (1987), Manos Hadjidakis
	2	Introduction to flow: legato	Gradual moderate intensity and limp and torso mobilization		Specific warm-up and introduction to contemporary dance	“By the Sea,” *Eternity and a Day* (1998), Eleni Karaindrou
	3	Storytelling: “The Outside the Window Travel”	Introduction to spine twist movements and arm waves		Aesthetics, fantasy stimulation, and visualization	“The Young Rallou” (remastered 2004), *30 Nocturnes* (1987), Manos Hadjidakis
	4	Introduction to upper limb velocity: staccato	Tapping movements, rotational arm movements, gestures, and visual contact		Coordination, diadochokinesia, and group dynamics	“The River,” *The River* (1959), Manos Hadjidakis
	5	Story telling: “The Ocean Dive”	Torso and upper extremities “waving”		Fantasy and imagination stimulation and visualization, successive movement	“Two,” *Two* (2006), Konstantinos Vita
	6	Limb and torso mobilization	“Flex point”		Coordination and seated posture	“Who Pays the Ferryman,” *Who Pays the Ferryman* (1977), Yannis Markopoulos
	7	Storytelling: “High Five”	Arm waves, stretch side to side, high five in pairs interaction, and spine rotation		Social interaction, spine mobilization, and group dynamics	“When the Clouds Come,” *Gioconda’s Smile* (1965), Manos Hadjidakis
	8	Introduction to velocity: staccato	Rhythmical walking on the spot and transition of weight		Rhythm, speed switching, and coordination	“Rain,” *Gioconda’s Smile* (1965), Manos Hadjidakis
	9	Storytelling: “Opa”	Staccato-and-legato body movements		Social interaction, speed switching, and movement dynamics (staccato-legato transitions)	“I Hartaetoi” (remastered), *I Gitonia Ton Aggelon* (2004), Mikis Theodorakis
**2 (supported by chairs—standing position)**						
	10	Ballet barre exercises	“Pliés relevés” in 1st and 2nd foot position, coordinated arms and legs movements, and “port de bras” movements		Introduction to basic ballet steps, balance, coordination, and posture	Movement 9, *Mythodea—Nocturne* (2019), Vangelis Papathanasiou (Vangelis)
	11	Introduction to the standing coordinated movement	Transition of weight side to side, on-spot walking, coordinated limb movements		Coordination, rhythm, and balance	“Kemal (O Mithos Tou Sevah),” *30 Nocturnes* (1987), Manos Hadjidakis
	12	“Sirtaki”	Transition of weight side to side, on-spot running		Introduction to basic *sirtaki* steps, coordination, balance, and stamina	“The Original Sirtaki Zorbas,” *Zorba the Greek* 1964, Mikis Theodorakis
**3 (nonsupported standing position)**						
	13	“Zeibekiko”	Forward-backward-side transfer of weight, turns, and free improvisation		Rhythm, creativity, and introduction to basic *zeibekiko* steps	“Evdokia’s Zeibekiko,” *Evdokia* (1972), Manos Loizos
	14	“La Révérence”: the goodbye section	Movement in a circle and one-leg balance positions with open and closed eyes		Balance, group dynamics, cooldown, and finish of the class	“A Stroll to the Moon,” *30 Nocturnes* (1987), Manos Hadjidakis

#### Strategies for Improved Adherence to Interventions

Although it has already been shown that dance interventions have higher adherence rates and lower attrition rates than other types of exercise in people with PD [49,50], we have implemented several actions within a more patient-centered and culturally tailored approach, as described in subsequent sections, to further increase patient continued participation.

#### Patient-Centered Dance Therapeutic Intervention

During the last decades, biomechanical-based therapeutic models in physiotherapy [51,52] have been progressively replaced by more “embodied” therapeutic approaches, in which each diversity and difference is accepted in a more inclusive attitude, which considers patient’s bodily and movement image [53]. This approach can be seen as part of a more personalized patient-centered physiotherapeutic approach, which is considered as a practical application of the biopsychosocial model [54,55] in terms of integrating the “patient world” in a comprehensive, empathic, and individualized tailored approach [56], and has been already used by physiotherapists, as a more holistic therapeutic approach in neurological patients [57]. The above model takes into serious consideration patients’ preferences, needs, and values [58], while it seems to enhance power, responsibility, and therapeutic alliance [59], and relates to better outcomes and QoL in patients with PD [60].

As there are many models and conceptualizations around patient-centeredness in physiotherapy [61], we proposed an 8-number domain of patient-centeredness in therapeutic dance interventions for PD patients with PD (Textbox 1, adapted from the studies by Stewart [55], Brown et al [62], Little et al [63], Stewart [64], Langendoen [56], Dukhu et al [65], and van der Eijk et al [58]), which was adapted from the patient-centeredness approach in medicine [55,62-64], the aspects of patient-centeredness in physiotherapy [56], the person-centered physiotherapeutic management of long-term conditions [65], and the patient-centered health care aspects for patients with PD [58].

The procedures that will be performed during our study according to this proposal are outlined in Textbox 2.

**Domains of patient-centeredness in therapeutic dance interventions for patients with Parkinson disease.**

1. A multidimensional and empathic assessment by a trained therapeutic dance health care professional with experience in Parkinson disease for a holistic understanding of the patients’ issues, concerns, preferences, and need for tailored information
2. A comprehensive planning and explaining of the physical assessment and the therapist’s hypotheses
3. Mutual agreement between patient and therapist on the multidimensional characteristics of the patient’s tailored issue after a holistic and valid assessment and interdisciplinary scientific collaboration
4. Mutual agreement between patient and therapist on the therapeutic intervention’s goals
5. Building of a high-confidence environment, accompanied by emotional support, empathy, and respect, between patient and therapist for enhancing the patient’s levels of self-responsibility, self-management, and independence
6. Treatment adaptations and modifications during possible circumstances differentiation
7. Individualization of exercises, dance combinations and techniques, and teaching methods
8. Sufficient and realistic use of resources and time and continuous health care accessibility

**Procedures that will be performed during the study.**

1. The assessment is structured, for research reasons; it is characterized by empathy, and includes multiple movement and nonmovement aspects of Parkinson disease (PD), giving a full informative description of the study’s procedures and goals to participants by the main researcher (ME), who is a specialized neurological diseases physiotherapist, and an adapted physical and dance educator. Although ME is responsible for the whole communication with patients, assessments before and after each research period will be performed by an independent researcher who is specialist in the use of the specific tools to eliminate possible detection bias. Part of the assessment (Movement Disorder Society–sponsored revision of the Unified Parkinson’s Disease Rating Scale [MDS-UPDRS] and Montreal Cognitive Assessment [MoCA] tests) will be performed by specialized neurologists of the Eginition Neurological Outpatient Clinic (domains 1 and 3).
2. Each step of the assessment procedure before and after each research period is explained; the necessity that each patient gives continuous feedback about possible adverse events is emphasized (domain 2).
3. Translated and validated scientific tools are used, and a continuous multidisciplinary scientific collaboration between physicians, nurses, and physiotherapists is ensured as part of this study (domain 3).
4. Therapeutic intervention goals were based on past research; however, personal patient needs were adapted in the dance combination’s design, based on our past pilot study [45], while individual music preferences were considered for the dance intervention’s design [66] (domain 4).
5. A high-confidence environment, accompanied by emotional support, empathy, and respect between participants and the physiotherapist, will be ensured (domain 5).
6. Although the early- to midstage severity stage of PD of participants usually does not demand specific modifications for each participant, due to the related motor independence with low fall risk [40], we are prepared to adapt our dance intervention following possible individual circumstances (domain 6).
7. Dance for Parkinson’s Disease (DfPD) classes are generally characterized by supportive instruction in a way that participants feel free to perform each dance combination in their individual level of functionality [67]; thus, inclusion, adaptability, and accessibility to all are promoted during each dance class [68] (domain 7).
8. The dance intervention is being conducted in a facility that is offered for free. Classes will be conducted solely by the main researcher (ME), while the trial will be partially funded by a charitable association, and continuous accessibility of patients to the research team will be provided (domain 8).

#### Culturally Tailored Therapeutic Dance Intervention

Most nation states were created during the 19th century, when cultural elements were used as potent symbols to serve each national identity [69], such as music and dance [70]. Dance has been used as a powerful tool in the creation of national identity [71] and as a national symbol that seems to emotionally empower members of a community [72]. Particularly in Greece, events involving dance are used for entertainment, socializing, and escape from everyday routine, as well as a sign of well-being that underpins the Greek national identity [73,74].

Dance is a physical activity in which the basic tool is the human body and its purposefully selected movements in a potentially varied rhythmical and cultural context [8]. Dance can be defined as ethnic, where movement types and motivation agree with the way of life and socially accepted norms of a period, and theatrical, where movement patterns are consciously designed to be shown to an audience that is not necessarily familiar with its context [75], even considering that every dance in any context contains ethnic elements [76]. Each person, even unconsciously, dances, bringing to bear all their past intra- and interpersonal experiences and cultural values within the specific socioeconomical environment they live [77], we decided to examine the effect of our dance intervention practice taking into consideration the cultural background of the targeted individuals, who in our case were Greek patients with PD.

DfPD is a dance intervention specially designed for patients with PD, which integrates different dance genres, encourages dance exploration, and focuses on dancing individually and in groups rather than in pairs [45]. For culturally adjusting this intervention to our population, we proceeded with several actions, as noted subsequently. First, we adopted the term “culturally tailored,” instead of “culturally adjusted,” as it has been already proposed in past research and it refers to the total sum of adaptations that must be done in terms of content, symbolisms, and strategies in an intervention to best suit specific cultural characteristics, and thus positively affect health behavior and cultural expression [78].

To incorporate Greek culture elements into our dance intervention, we focused on both surface structure (culturally matched music and dance movements or steps) and deep structure (social and historical influences) as prerequisites for feasibility and efficacy or impact of the dance intervention accordingly [79]. Although collecting participant needs and experiences through exploratory focus groups may better inform both the surface and deep structure of the intervention [79], we based our intervention on past research and participants’ music preferences [66], as described subsequently, to reduce the cost and duration of the study (domain 8, Textbox 1).

All the 14 selected music pieces were composed by Greek artists, and they have been chosen for their capability to serve the idea of Greekness or “Hellinikotita” and being well known in most of the Greek population. While Greek folk dances can be used as a therapeutic tool for older adults at risk of neurodegeneration [80], and they seem to improve functional and psychological characteristics leading to better outcomes in terms of QoL in patients with chronic diseases [81], 2 of the earlier mentioned dances (tracks 12 and 13) were selected for the aforementioned reasons. Furthermore, they seem to have several semiotic cultural references; thus, they may involve symbolic signals for the Greek population [82,83]. The explanation for each music selection is noted below (Table 5).

**Table 5:** Explanation for each musical selection in terms of cultural tailoring.

Tracks	Composer	Explanation for each music selection
1, 3, 4, 7, 8, 11, and 14	Manos Hadjidakis	Manos Hadjidakis was a well-known Oscar-winning Greek composer whose work led to the development of a musical language combining popular musical elements directly connected with the Greek cultural memory [84].
9, 12	Mikis Theodorakis	Mikis Theodorakis’ work, along with that of Hadjidakis, influenced the idea of Greekness or “Hellinikotita” in the music of the 20th century, while he was characterized by his political influence on late 20th century in Greece [85]. Especially, the 12th track refers to the internationally well-known Greek folk dance “syrtaki,” which has been the most famous Greek melody since its birth in 1964, and as part of the film “Zorba the Greek” has been included as a musical semiosis to the Greek cultural identity [82,86].
2	Eleni Karaindrou	Eleni Karaindrou is a Greek artist established for her works in the cinema [87]. The music piece has been chosen from the film *Eternity and a Day* by Theo Angelopoulos, a movie that influenced Greek filmography of the past century and managed in an artistically abstract way to reconstruct the history of Greece, the Balkans, and Europe in a context full of historical and political allegories [88].
13	Manos Loizos	This track refers to the most well-known music that accompanies the “zeibekiko” dance (“Evdokia’s Zeibekiko”), a mostly improvisational Greek folk dance directly connected with “rebetiko” culture, and which, from 1980 onwards, has been defined as a pan-Hellenic semiotic dance, leading someone to nonverbally express their emotions [83].
6	Yannis Markopoulos	Yannis Markopoulos is an internationally well-known Greek composer. The selected musical piece was composed for the British Broadcasting Corporation (BBC) television series “Who Pays the Ferryman?” a drama series with great success in Great Britain, Europe, Canada, and Australia [89].
5	Konstantinos Vita (known as K.BHTA)	K.BHTA is the founder of the well-known Stereo Nova Greek electronic music band (1992-1996), while the selected music piece was produced to accompany the dance performance 2 of Dimitris Papaioannou (2006), an internationally well-known Greek choreographer, and artistic director of the opening and closing ceremonies of the 2004 Athens Summer Olympic Games.
10	Evangelos Odysseas Papathanassiou or Vangelis Papathanasiou (known as Vangelis)	Vangelis was a well-known Oscar-winning Greek composer, and the selected music piece is part of the internationally well-known music album *Mythodea—Music for the NASA Mission: 2001 Mars Odyssey*.

#### Music Preferences

People seem to develop musical identities from an early age in the context of constructing a more general self-identity [90], either as individuals (personal identity) or as members of larger social groups (social identity) [91,92]. Music preferences play a key role in both personal and social parameters of self-identity by participating in the construction of (1) self-image, (2) self-definition, (3) sense of belonging and acceptance by a certain social group, and (4) specific personal values’ reflection [66].

In the “musical preferences” scale by Gardikiotis and Baltzis [66], 24 music genres are included on a 6-point scale (0=I do not know this music genre and 1=I do not like it at all, to 5=I like it very much) [93], and divided into 5 factors: (1) “sentimental and sensational,” (2) “sophisticated and complex,” (3) “nonmainstream dissonant,” (4) “established rebellious,” and (5) “native-Greek traditional.”

During the enrollment phase (Table 3), music preferences of all the participants were registered, and taking in serious consideration the strong music preference frequency (“I like it very much”) of most participants (>50%), we compiled the list of selected music titles of the intervention (Table 4). As it is shown in Table 6, the first 4 categories of strong music preferences frequency were formed by music genres of the “established rebellious,” which includes Greek art popular (*entechno*), and “native Greek traditional” scale’s factors, which includes *rebetika*, *laika* (Greek urban popular), and Greek folk (traditional) music.

**Table 6:** Strong music preference frequency (“I like it very much”) based on a 6-point scale.

Music genre	Frequency “I like it very much,” n (%)
Greek art-popular (*entechno*)	24 (60)
Greek urban-popular (*laika*)	24 (60)
*Rebetika*	24 (60)
Greek folk (traditional)	23 (57.5)
Classical	19 (47.5)
Greek rock	18 (45)
Greek pop	18 (45)
Pop (contemporary and Western-style)	18 (45)
Jazz	18 (45)
Rock ‘n’ roll	18 (45)
Blues	17 (42.5)
Greek *laika* pop	14 (35)
Rock	14 (35)
World (ethnic) music	13 (32.5)
Reggae	9 (22.5)
Soul or rhythm and blues	8 (20)
Hard rock or metal	6 (15)
Greek rap or hip-hop	4 (10)
Rap or hip-hop	3 (7.5)
Funk	3 (7.5)
Punk	3 (7.5)
House	3 (7.5)
Alternative	2 (5)
Trance	1 (2.5)

#### Criteria for Discontinuing or Modifying Allocated Interventions

Participants are free to withdraw from the study at any time and for any reason. As it has been previously mentioned, according to the patient-centered approach that we follow, we have been prepared to adapt our dance intervention following possible differentiation in circumstances (domain 6, Textbox 1). DfPD classes are generally characterized by supportive instruction in a way that participants feel free to perform each dance combination at their individual level of functionality [67]; thus, any personal modification or interpretation is always encouraged to be performed by each participant (domain 7, Textbox 1).

### Outcomes

#### Primary Outcomes

Patients’ QoL before and after each research period will be assessed by the change in total score of the Greek version [94] of Parkinson’s Disease Questionnaire-8 [95], which is a 0-to-100-point scale with a higher score indicating worse QoL.

#### Key Secondary Outcomes

The expected key secondary outcomes are outlined in Textbox 3.

**Key secondary outcomes.**

**Safety (occurrence of adverse events and serious adverse events)**

The occurrence of adverse events such as possible falls, injuries, muscle soreness, and excessive fatigue [96] will be assessed by recording after the end of the program, as has been done in our past pilot clinical study [45], for each participant. The following are considered as serious adverse events [96,97], and they will be recorded after the end of the program: death, threat to life, hospitalization, persistent or significant disability or incapacity, and necessity of surgical operation for any of the aforementioned events.

**Feasibility**

Adherence rates: an adherence rate of ≥70% is considered as high in older patients with functional limitations [98], and it will be used to assess adherence to each dance intervention following the appropriate formula after the end of the program:

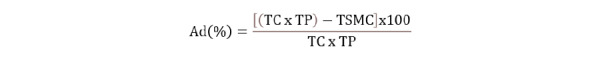

Attrition rates: an attrition rate of ≤15% is considered as acceptable by the Physiotherapy Evidence Database Scale [99], and will be used to assess attrition to each dance intervention following the appropriate formula after the end of the program:

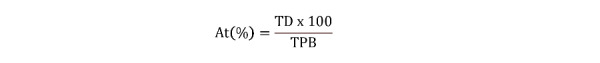

Recruitment rates: for the assessment of the recruitment rates, a target of up to 5 months for 40 participants will be accepted according to past studies [45,100] after the end of the program.

**Patient satisfaction**

Willingness to continue the program after the intervention: a verbal statement for continuing the program after the end of the intervention in a 6-point Likert scale (0=I don’t know if I’d like to continue the program, 1=I don’t want to continue the program at all, 2=I don’t want to continue the program, 3=I might want to continue the program, 4=I want to continue the program, and 5=I want to continue the program very much) after the end of the program.

Advantages and disadvantages of the program: there will be a total of 2 open questions: (1) Could you please list all the positive and negative points of the in-person Dance for Parkinson’s Disease (DfPD) intervention? and (2) Could you please list all the positive and negative points of the online DfPD intervention? These will be asked in a one-to-one interview of each participant by the principal investigator (ME) after the end of the program for the qualitative recording of advantages and disadvantages of each dance intervention from the patient’s perspective.

#### Secondary Outcomes

The other expected secondary outcomes are outlined in Textbox 4.

**Other secondary outcomes.**

**Fatigue**

- Parkinson Fatigue Scale-16 (PFS-16): fatigue will be assessed before and after each research period by the change in total score of the Greek version [101] of PFS-16 [102], which is a 1- to 5-point scale with a higher score indicating more fatigue.
- Modified Fatigue Impact Scale: fatigue will be also assessed before and after each period by the change in total score of the Greek version [103] of the Modified Fatigue Impact Scale [104], which is validated in the population with Parkinson disease (PD) [105] 0- to 84-point scale, with a higher score indicating more fatigue.

**Depressive symptoms**

- Depressive symptoms will be assessed before and after each research period by the change in total score of the Greek version [106,107] of the depression subscale score of Depression, Anxiety, and Stress Scale-21 (DASS-21) [108], which is a validated in the population with PD [109] 0-to-42-point scale with 0 to 9 indicating no symptomatology, 10 to 12 indicating mild symptomatology, 13 to 20 indicating moderate symptomatology, 21 to 27 severe symptomatology, and 28 to 42 extremely severe symptomatology.

**Stress**

- Stress will be assessed before and after each research period by the change in total score of the Greek version [106,107] of the stress subscale score of DASS-21 [108], which is validated in the population with PD [109] 0-to-42-point scale, with 0 to 10 indicating no stress, 11 to 18 indicating mild stress, 19 to 26 indicating moderate stress, 27 to 34 severe stress, and 35 to 42 extremely severe stress.

**Anxiety**

- Anxiety will be assessed before and after each research period by the change in total score of the Greek version [106,107] of the anxiety subscale score of DASS-21 [108], which is validated in the population with PD [109] 0- to 42-point scale, with 0 to 6 indicating no anxiety, 7 to 9 indicating mild anxiety, 10 to 14 indicating moderate anxiety, 15 to 19 severe anxiety, and 20 to 42 extremely severe anxiety.

**Sarcopenia**

- Sarcopenia risk will be assessed before and after each research period by the change of total score of the Greek version [110] of Strength, Assistance in walking, Rise from a chair, Climb stairs, and Falls Scale [111], which is a 0 to 10 self-reporting screening tool of five 0- to 2-point components (strength, assistance walking, rise from a chair, climb stairs, and number of falls) with 0 to 3 indicating no risk for sarcopenia, and ≥4 indicating risk of sarcopenia.
- Sarcopenia status will be assessed before and after each research period by the change of European Working Group on Sarcopenia in Older People criteria [112], as has been previously assessed in the PD population [113]. According to the European Working Group on Sarcopenia in Older People, sarcopenia is (1) probable in an individual with low muscle strength (hand grip strength [HGS] for men: <27 kg and women: <16 kg), (2) confirmed in an individual with the above criteria combined with low muscle quantity (appendicular skeletal muscle mass index for men: <7 kg/m^2^ and women: <5.5 kg/m^2^), and (3) severe in an individual with the 2 above criteria combined with low physical performance (4 m usual walking speed test≤0.8 m/s). HGS will be assessed by a calibrated digital handheld dynamometer, appendicular skeletal muscle mass index by a bioelectrical impedance analysis equipment, and physical performance by the4-m usual walking speed test.

**Frailty**

- Frailty will be assessed before and after each research period according to the most often used frailty scale in PD [4] frailty phenotype (FP) status [114], in which 5 criteria have to be assessed: (1) unintentional weight loss >5 kg the last year, (2) weakness (HSG for men: <27 kg and women: <16 kg), (3) slow walking speed (10-m usual walking speed test ≤0.8 m/s), (4) exhaustion (PFS-16 total score ≥3, which indicates moderate to severe fatigue), and low levels of physical activity (a total score <600 MET/min/week^-1^ indicates inactivity in the Greek version of International Physical Activity Questionnaire-Short Form [115]). The FP score ranges from 0 to 5 (1 point for each criterion; 0: best score to 5: worst score), and defines as frail an individual with a score of ≥3, as prefrail an individual with a score of 1 to 2, and as nonfrail or robust an individual with a score of 0.

**Balance**

- Balance will be assessed before and after each research period by the Greek version [116] of Berg Balance Scale [117], which is a 14-item validated in PD [118] scale with a minimum score of 0, and a maximum score of 56, while lower scores indicate worse balance (0-20: high fall risk, 21-40: medium fall risk, and 41-56: low fall risk).

**Cognitive functions**

- Cognition will be assessed before and after each research period by the Greek version [119,120] of Montreal Cognitive Assessment (MoCA) [121], which is a 0- to 30-point scale with lower scores indicating cognitive impairment.

**Movement and nonmovement PD symptoms**

- Movement and nonmovement PD symptoms will be assessed before and after each research period by the Greek version of Movement Disorder Society–sponsored revision of the Unified Parkinson’s Disease Rating Scale (MDS-UPDRS) [122], which is a 4 parts scale. Part I assesses nonmotor experiences of daily living (13 items, score range: 0-52), part II assesses motor experiences of daily living (13 items, score ranges: 0-52), part III assesses motor signs (33 items, score ranges: 0-132), and part IV assesses motor complications, namely dyskinesias and motor fluctuations (6 items, score ranges: 0-24). Each item score ranges from 0 (normal) to 4 (severe), and higher scores indicate a greater impact of PD signs.

**BMI**

- BMI will be assessed before and after each research period by the score from the equation kg/m^2^, with <18,5 indicating underweight, 18.5 to 24.9 indicating normal weight, 25 to 29.9 indicating overweight, and ≥30 indicating obesity (3 obesity subcategories exist: Class I scores of 30-34, Class II scores of 35-39, and Class III scores of ≥40) [123].

### Sample Size

A prospective, nonrandomized, uncontrolled, open-label pilot study has already been conducted in a Greek population of 16 patients with early- to midstage PD, showing that in-person DfPD is safe and feasible, and can improve QoL, as well as several movement and nonmovement PD symptoms [45]. According to the results of the above pilot clinical trial for our primary outcome (QoL), the Cohen equations for sample size calculation [124], and considering the power of the study equal to 80% and the level of statistical significance of the controls equal to the conventional limit of statistical significance (α=.05), a power analysis was applied with the a priori formula for the *F* test statistical control through analysis of variance with repeated measures using G*Power software (version 3.1) [125].

Taking in consideration that the number of experimental groups will be 3, the number of repeated measurements for each participant will be 6, and the average correlation coefficient between repeated measurements will be 0.64, it was estimated that for this study, which has a 1:1:1:1:1:1 distribution ratio of participants to the groups, at least 6 participants are required in each group (18 in total). On the basis of the equations that adjust the sample size to the expected dropout rate [126], which is expected to be up to the 15% acceptable value, based on the Physiotherapy Evidence Database Scale [99], the minimum sample size required to achieve the study’s primary end point (QoL) is 21 participants.

### Recruitment

Recruitment started 6 months before the starting date of the program via relevant printed information material and health care practitioners (neurologists and nurses) of the Eginition University Hospital Neurological Outpatient Clinic. All study procedures have been discussed face-to-face individually with each potential participant before screening for eligibility by the primary researcher. The primary researcher (ME) has obtained written informed consent from the potential clinical trial participants before the study’s inclusion.

### Assignment of Interventions: Allocation

A block randomization will be conducted after the completion of the first baseline assessment (Figure 1) in equal ratio (1:1:1:1:1:1) to 1 of the 6 study sequences (ABC, BCA, CAB, ACB, BAC, or CBA), as further shown in Table 1 [127]. Concealment is ensured by keeping study material in sealed, opaque envelopes and by using paper assignment. After baseline 1 assessment completion (Figure 1), each participant will be randomized to 1 of the 6 study sequences by the data analyst, and the document with the final groups’ formation will be delivered in a sealed envelope to the principal investigator, who is the responsible for participants’ enrollment.

### Data Collection and Management

All assessments will be conducted face-to-face by an independent investigator to eliminate possible detection bias, except of the Movement Disorder Society-Unified Parkinson’s Disease Rating Scale and Montreal Cognitive Assessment, which will be conducted face-to-face by specialized neurologists of the Eginition University Hospital Neurological Outpatient Clinic.

Participants are free to quit at any time if they wish to discontinue the study. In that case, reasons for withdrawal will be recorded, if this is possible. Participant’s enrollment will be followed by the research team’s assistance during the entire study period, including scheduling of data collection appointments, reminding of intervention’s appointments, and communicating the starting time of each research period.

Data, including consent forms, completed paper-based data files, and study notes, will be securely stored in the main investigator’s office, which will be locked. Raw data will be encrypted and stored digitally on a local computer with access rights to the main investigator. All original data will be archived for 5 years after the completion of the research and then destroyed, according to local regulations. All written data will be stored in a locked cabinet of the main investigator’s locked office for 5 years and then destroyed. Digital data will be stored on a password-protected local computer.

### Statistical Analysis

The primary analysis will evaluate the between group differences in total score of the Greek version of Parkinson’s Disease Questionnaire-8 after each research period. Mean (SD) or summary statistics appropriate to the distribution of the QoL total score will be reported by study group (in-person DfPD vs online DfPD vs control). Study groups will be compared in terms of QoL using mixed model repeated measures considering the correlated data from the same patient [128]. Mixed model repeated measures are robust in small sample size studies; thus, a decreased risk of type II errors occurs [129]. For all secondary end points, summary statistics will be tabulated by study group, and study groups will be compared with a statistical method appropriate to the type of the end point (eg, continuous and categorical end point). Bonferroni correction will be used to account for multiple comparisons of secondary outcomes.

A secondary analysis of the primary end point will adjust for those prerandomization variables which might reasonably be expected to be predictive of favorable QoL total score. Any deviations from the protocol will be recorded and agreed by the investigators. Missing data will not be imputed neither for primary nor secondary analyses, and no interim analyses will be conducted in this study. All the *P* values are 2-sided at an α level of .05. Analyses will be conducted with SPSS (version 20.0; IBM Corp) and R (version 4.4.1; R Foundation for Statistical Computing).

## Results

The study is a 3-arm crossover and open-label randomized controlled trial (in-person DfPD vs online DfPD vs control), and was partially funded in September 2023. The first participant was enrolled in April 2023. The trial is currently ongoing, and will conclude in September 2024. A total of 47 patients were enrolled during the recruitment period, of whom 7 have been excluded (Figure 1). A total of 40 patients were randomized equally to the study’s 6 sequences to undergo sixteen 60-minute culturally tailored DfPD classes both in-person and online during a 10-month experimental period following a patient-centered approach. The data analyst will analyze all the data after the final participant exists the study. We expect that by the second quarter of 2025, the study’s results will have been published.

## Discussion

### Principal Findings

This is a 3-arm crossover and open-label randomized controlled trial (in-person DfPD vs online DfPD vs control), which aims to evaluate the efficacy, safety, and feasibility of a patient-centered and culturally tailored DfPD intervention delivered both in-person and online as a complementary therapy for a series of movement and nonmovement PD symptoms, as well as for QoL, sarcopenia, and frailty of Greek patients with PD.

Frailty is a state most common in older people, characterized by high vulnerability to acute stressors, such as falls, which are associated with increased hospitalization and mortality risk as well as disease severity or progress [130]. Patients with PD, accompanied by sarcopenia and frailty, seem to have lower cognitive function, movement performance, and QoL; recurrent falls; and higher fatigue, orthostatic hypotension, and inpatient mortality. Physical exercise programs seem to reduce or prevent the risk of both conditions; thus, they may improve the QoL of patients with PD [2,4,6,131]. The possible effect of DfPD on sarcopenia and frailty of Greek patients with early-to-midstage PD has not been examined. Furthermore, there is no controlled study investigating any possible differential effect of in-person and remote delivery of the aforementioned program on the QoL of patients with PD, fatigue, depressive symptoms, stress, anxiety, sarcopenia, frailty, balance, cognitive functions, movement and nonmovement PD symptoms, and BMI. We expect that our results will aid in the implementation of well-organized patient-centered and culturally tailored dance programs for patients with PD, thus providing reliable evidence for better recommendations for clinical practice.

### Conclusions

The results of this study are expected to show the possible differential effect of a patient-centered and culturally tailored DfPD intervention, delivered in-person versus online, on several movement and nonmovement symptoms, as well as on QoL, sarcopenia, and frailty in people living with PD in Greece.

## Abbreviations

CONSORT: Consolidated Standards of Reporting Trials
DfPD: Dance for Parkinson’s Disease
PD: Parkinson disease
QoL: quality of life
UPGRADE-PD: Upbeating Greek Application of Dance in Parkinson’s Disease
